# *Neocaridinafonticulata*, a new land-locked freshwater shrimp from Hengchun Peninsula, Taiwan (Decapoda, Caridea, Atyidae)

**DOI:** 10.3897/zookeys.817.29332

**Published:** 2019-01-15

**Authors:** Hsi-Te Shih, Yixiong Cai, Yuh-Wen Chiu

**Affiliations:** 1 Department of Life Science and Research Center for Global Change Biology, National Chung Hsing University, 250, Kuo Kuang Road, Taichung 402, Taiwan National Chung Hsing University Taichung Taiwan; 2 National Biodiversity Centre, National Parks Board, 1 Cluny Road, Singapore 259569, Republic of Singapore National Biodiversity Centre Singapore Singapore; 3 Center for Research in Water Science and Technology, and Department of Hydraulic and Ocean Engineering, National Cheng Kung University, No. 1, University Road, Tainan City 701, Taiwan National Cheng Kung University Tainan Taiwan

**Keywords:** *
Neocaridina fonticulata
*, mitochondrial cytochrome oxidase subunit I, new species, morphology

## Abstract

A new species of land-locked freshwater shrimp, *Neocaridinafonticulata***sp. n.** (Atyidae), is described from Kenting, Hengchun Peninsula, Pingtung County, southern Taiwan. This new species can be distinguished from its congeners by rostrum structure, pereiopods, and male first and second pleopods. The molecular evidence of mitochondrial cytochrome oxidase subunit I (COI) also supports the establishment of a new species. This is the third endemic species of *Neocaridina* known from Taiwan.

## Introduction

The genus *Neocaridina* Kubo, 1938 is a group of small-sized shrimps with a land-locked habit, inhabiting in the middle and upper reaches of rivers in East Asia, with more than 30 species recorded (Liang 2004, De Grave and Fransen 2011, Shih et al. 2017). In the East Asian arc, three species have been reported from Taiwan, viz. *N.davidi* (Bouvier, 1904), *N.ketagalan* Shih & Cai, 2007 and *N.saccam* Shih & Cai, 2007; two species from the Ryukyus, viz. *N.ishigakiensis* (Fujino & Shokita, 1975) and *N.iriomotensis* Naruse, Shokita & Cai, 2006; and two species from the main islands of Japan, viz. *N.denticulata* (De Haan, 1844) and *N.ikiensis* Shih, Cai, Niwa & Nakahara, 2017, with several introduced species reported (Naruse et al. 2006, Shih and Cai 2007, Shih et al. 2017).

A recent survey of the species diversity of freshwater shrimps of Taiwan showed an undescribed species from southern Taiwan with different morphological characters compared to other known species of *Neocaridina*, which was supported by molecular evidence. This species is herein described as a new species, endemic to Taiwan Island, which brings the total number of Taiwanese species of *Neocaridina* to four.

## Materials and methods

Specimens of the genus *Neocaridina* examined in this study were collected from a spring in Sheding, Kenting, Hengchun Peninsula, Pingtung County, Taiwan and preserved in 70%–95% ethanol after collection. Some specimens were selected and illustrated with the help of a drawing tube attached to a Nikon stereo microscope (model SMZ 1000), and deposited in the Zoological Collections of the Department of Life Science, National Chung Hsing University, Taichung, Taiwan (**NCHUZOOL**) and the Zoological Reference Collection of the Lee Kong Chian Natural History Museum, National University of Singapore, Singapore (formerly the Raffles Museum of Biodiversity Research) (**ZRC**). Carapace length is abbreviated cl, and the mode refers to the most frequently number occurring. The rostral formula was counted based on all specimens available. The egg measurements were based on five eggs each from four ovigerous females (see material examined).

Sequences of mitochondrial cytochrome oxidase subunit I (COI) were obtained following the method described by Shih et al. (2017), with the primers LCO1490 and HCO2198 (Folmer et al. 1994). Sequences were obtained by automated sequencing (Applied Biosystems 3730xl DNA Analyzer), after verification with the complementary strand. Sequences obtained have been deposited in the DNA Data Bank of Japan (DDBJ) and were analyzed with other sequences published in Shih and Cai (2007) and Shih et al. (2017).

The best-fitting model for sequence evolution was determined by MrModeltest (version 2.2, Nylander 2005), selected by the Akaike information criterion (AIC). The obtained best model was HKY + G, and was subsequently used for the Bayesian inference (BI) analysis. The BI analysis was performed with MrBayes (version 3.2.3, Ronquist et al. 2012). The search was run with four chains for 10 million generations and four independent runs, with trees sampled every 1000 generations. The convergence of chains was determined by the average standard deviation of split frequency values below the recommended 0.01 (Ronquist et al. 2005) and the first 1150 trees were discarded as the burnin accordingly. The maximum likelihood (ML) analysis was conducted in RAxML (vers. 7.2.6, Stamatakis 2006). The model GTR + G (i.e., GTRGAMMA) was used with 100 runs, and found the best ML tree by comparing the likelihood scores. The robustness of the ML tree was evaluated by 1000 bootstrap pseudoreplicates under the model GTRGAMMA.

Other analyses, including the nucleotide composition, variable and parsimony informative positions, Kimura 2-parameter (K2P) distance (Kimura 1980) and p-distance between haplotypes were calculated using MEGA (version 5.2.2, Tamura et al. 2011).

## Systematic account

### Family Atyidae De Haan, 1849

#### *Neocaridina* Kubo, 1938

##### Neocaridina fonticulata sp. n.

Taxon classificationAnimaliaDecapodaAtyidae

http://zoobank.org/5F96C596-4AF1-43E1-971B-BA49C15D5E1F

**Figure 1.**
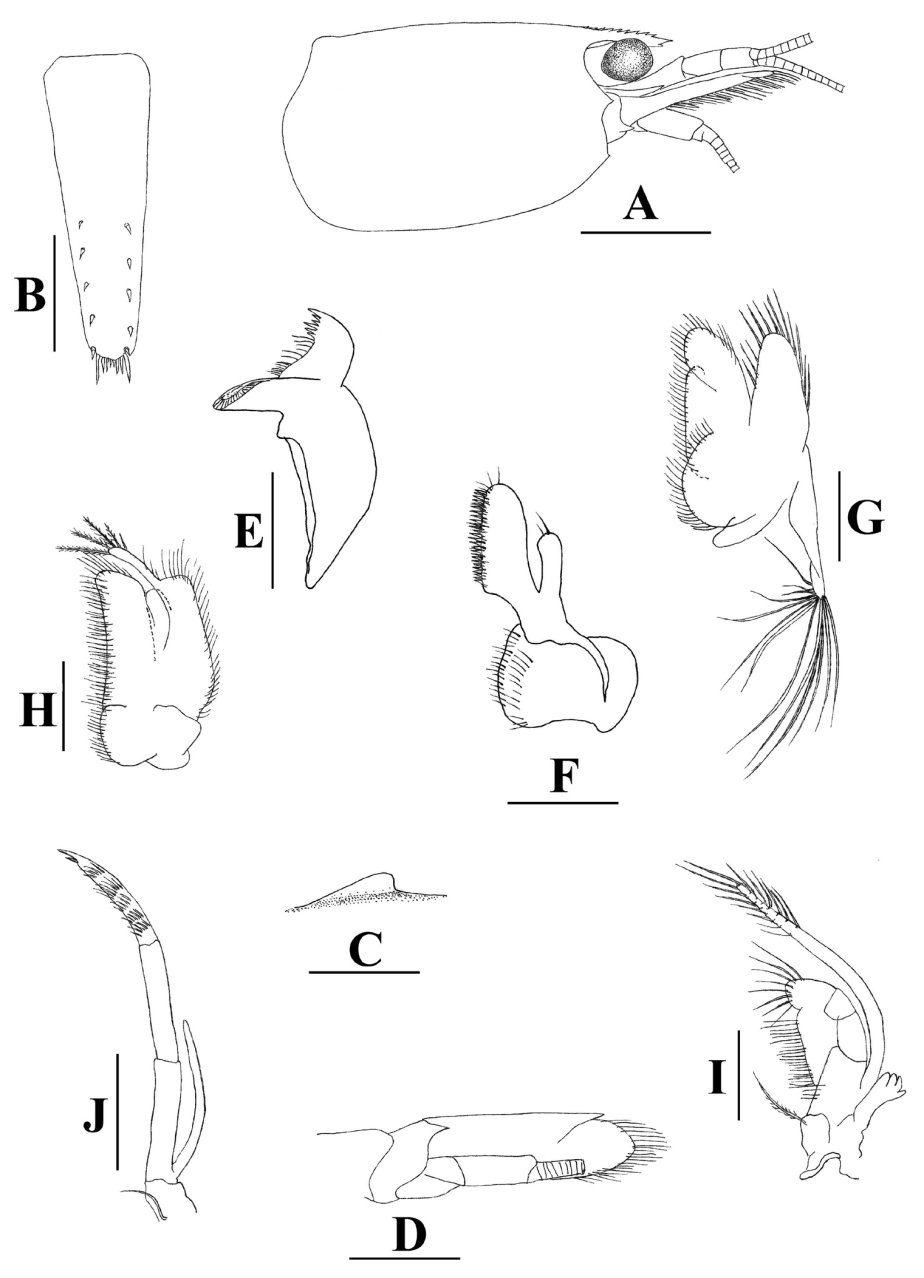
*Neocaridinafonticulata* sp. n.: **A** carapace and cephalic appendages, lateral view **B** telson, dorsal view **C** preanal carina, lateral view **D** right scaphocerite and antenna, ventral view **E** right mandible **F** right maxillula **G** right maxilla **H** right 1^st^ maxilliped **I** right 2^nd^ maxilliped **J** right 3^rd^ maxilliped. Scale bars: 1.5 mm (**A**); 0.5 mm (**B, E–J**); 1 mm (**C, D**) (male, cl 3.0 mm, paratype, ZRC 2018.1013).

**Figure 2.**
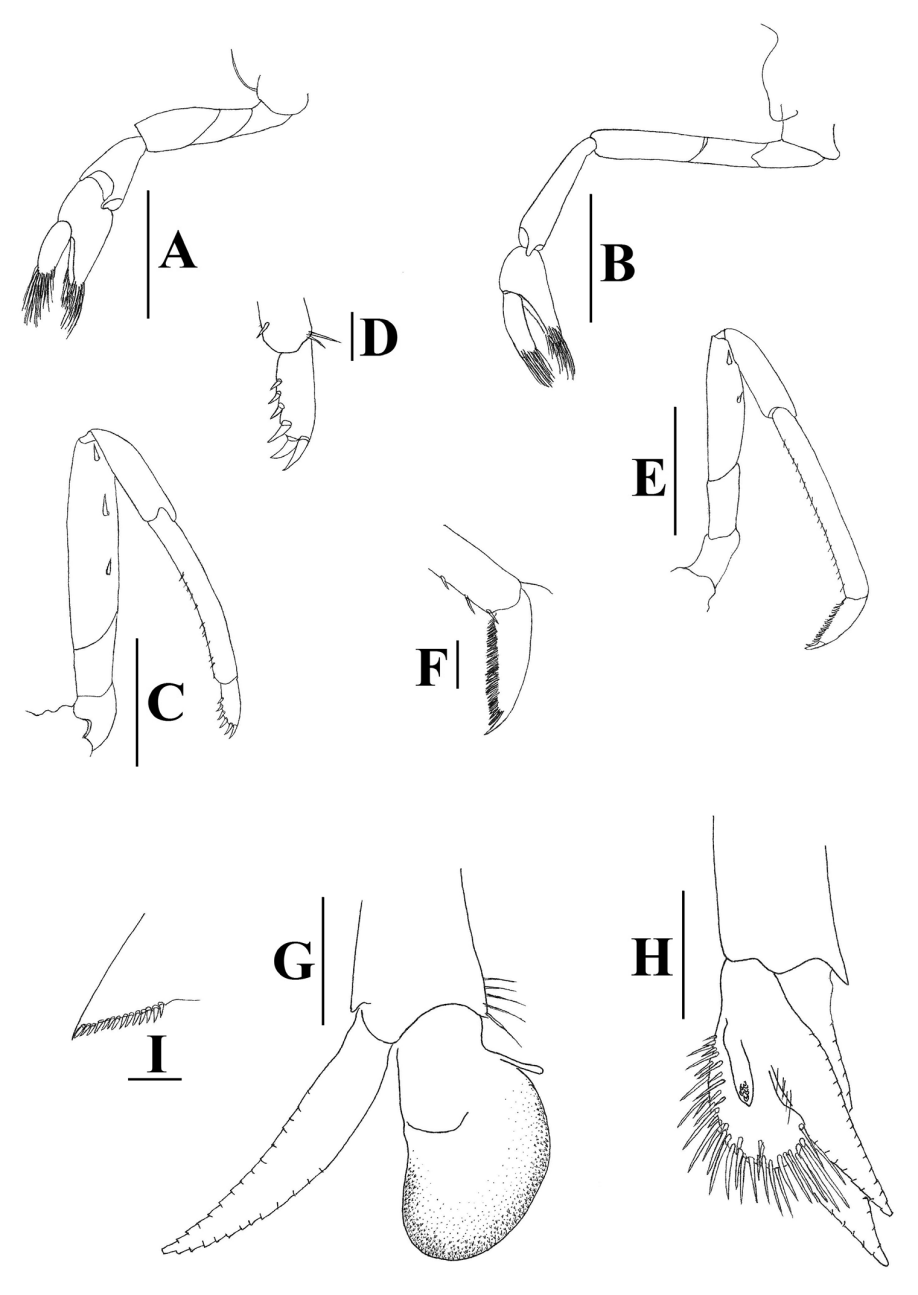
*Neocaridinafonticulata* sp. n.: pereiopods in lateral view. **A** right 1^st^ pereiopod **B** right 2^nd^ pereiopod **C** right 3^rd^ pereiopod **D** same, dactylus **E** right 5^th^ pereiopod **F** same, dactylus **G** right male 1^st^ pleopod, front view **H** right male 2^nd^ pleopod, internal view **I** diaeresis of left uropodal exopod. Scale bars: 1 mm (**A–C, E**); 0.2 mm (**D, F**); 0.5 mm (**G, H**); 0.2 mm (**I**) (male, cl 3.0 mm, paratype, ZRC 2018.1013).

**Figure 3.**
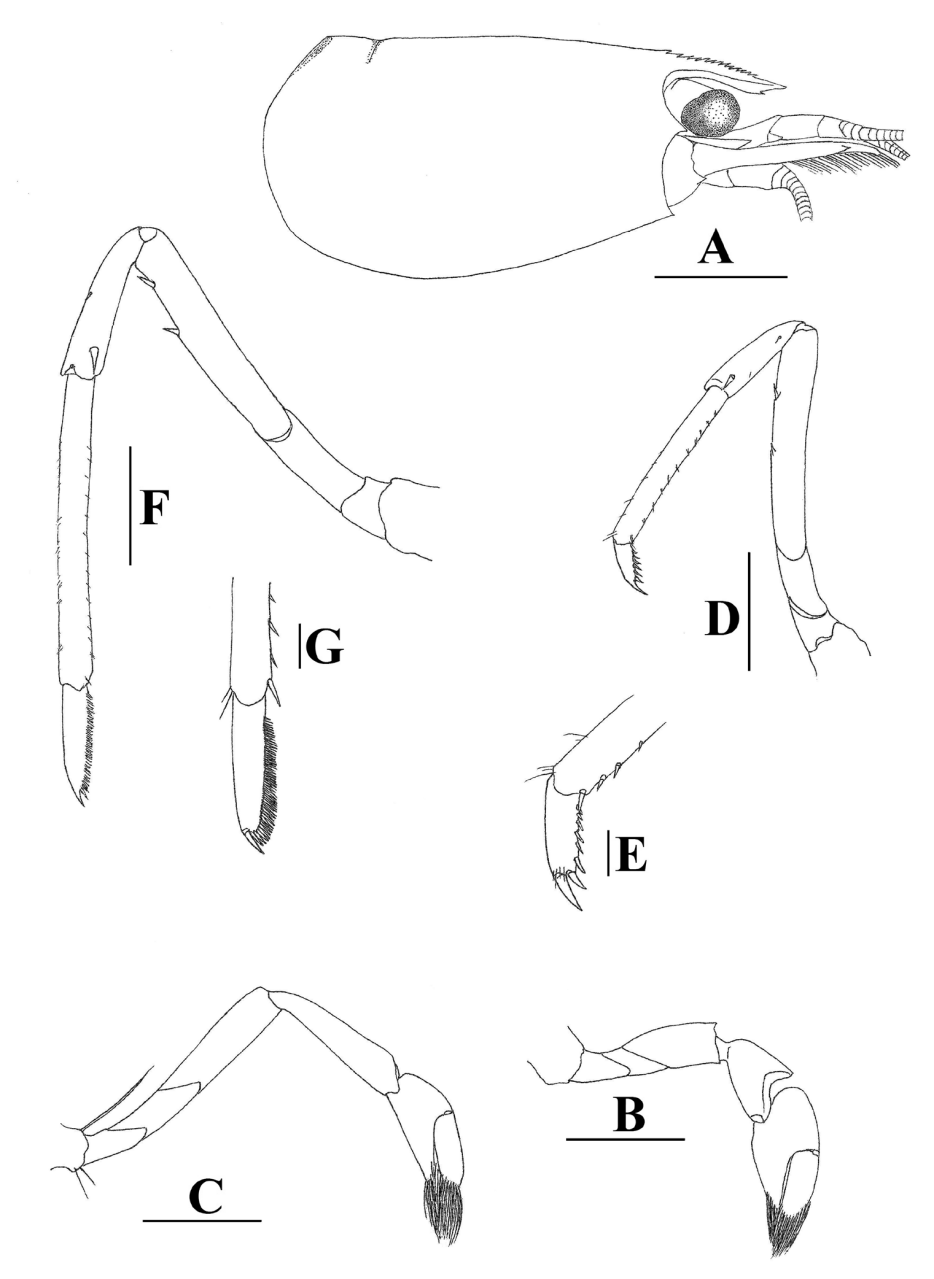
*Neocaridinafonticulata* sp. n.: **A** carapace and cephalic appendages, lateral view **B** right 1^st^ pereiopod **C** right 2^nd^ pereiopod **D** left 3^rd^ pereiopod **E** same, dactylus **F** left 5^th^ pereiopod **G** same, dactylus. Scale bars: 1.5 mm (**A**); 1 mm (**B–D, F**); 0.2 mm (**E, G**) (female, cl 3.8 mm, paratype, ZRC 2018.1013).

**Figure 4.**
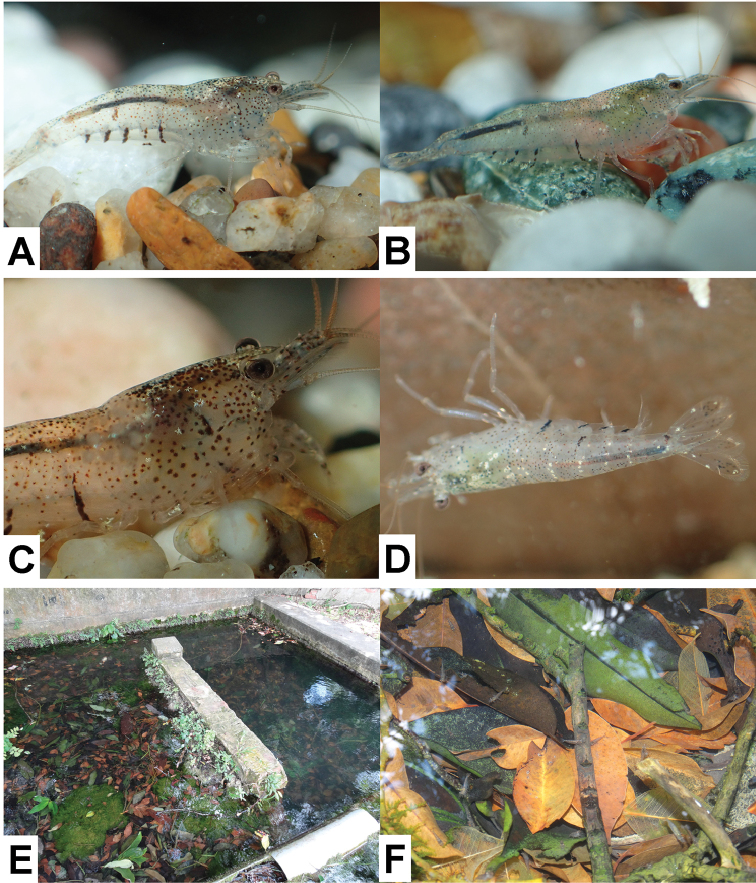
Live colouration of *Neocaridinafonticulata* sp. n. (**A–D**) and its habitat in Kenting, southern Taiwan (**E–F**). Specimens were collected from the type locality on 1 July 2015 and kept in aquarium for observation and photography.

Figures 1 , 2 , 3 , 4

###### Material examined.

Holotype: male, cl 3.4 mm, NCHUZOOL 15004, a spring at Sheding, Kenting, Pingtung County, Taiwan, 21°57'26.7"N, 120°48'35.5"E, elevation of 150 m, coll. H.-T. Shih and Y. C. Gan, 1 July 2015. Paratypes: 13 males, cl 2.5–3.3 mm, NCHUZOOL 15005, 5 females, cl 2.6–3.8 mm, 2 ovigerous females, cl 3.6–3.7 mm, NCHUZOOL 15006; 1 male, cl 4.2 mm, NCHUZOOL 15007; 1 male, cl 3.9 mm, NCHUZOOL 15008; 7 males, cl 2.7–3.3 mm, 2 females, cl 3.6–3.8 mm, 2 ovigerous females, cl 3.5–3.6 mm, ZRC 2018.1013, same collection data as for holotype. 1 male, cl 3.5 mm, 1 damaged specimen, cl 4.4 mm, NCHUZOOL 15009, Sheding, Kenting, Pingtung County, Taiwan, 5 May 2015, coll. Y. C. Gan.

###### Other material.

3 males, 9 females, NCHUZOOL 15010, Sheding, Kenting, Pingtung County, Taiwan, coll. Y. C. Gan, 5 May 2015. 3 males, 11 females, 2 ovigerous females, NCHUZOOL 15011, two damaged males, ZRC 2018.1014, same collection data as for holotype.

###### Comparative material.

*Neocaridinaikiensis*: 1 male, cl 4.6 mm, ZRC 2017.0960, 1 female, cl 5.1 mm, ZRC 2017.0961, 8 males, cl 3.0–5.4 mm, 8 females, cl 2.9–5.1 mm, ZRC 2017.0962, small stream at Kugiyama-hure, Gonoura Town, Iki City, Nagasaki Prefecture, Japan, coll. Yasuhiko Nakahara, 23 November 2015.

###### Diagnosis.

Rostrum short, straight, slightly sloping downwards, reaching mostly to end of 1^st^ segment of antennular peduncle, rostral formula 1–3+8–15/1–4. Pterygostomian margin armed with an indistinct spine. 1^st^ pereiopod carpus 1.2–1.5 × as long as high; chela 2.0–2.1 × as long as broad; fingers slightly longer than palm. 2^nd^ pereiopod carpus 1.1–1.2 × as long as chela, 3.9–4.3 × as long as high; chela 2.1–2.3 × as long as broad; fingers 1.3–1.4 × as long as palm. 3^rd^ pereiopod with propodus straight in females, slightly incurved in males, 2.7–3.0 × as long as dactylus; dactylus terminating in two claws, 4–6 accessory spines on flexor margin, strongly incurved in males. 5^th^ pereiopod propodus 2.7–2.8 × as long as dactylus, dactylus terminating in one claw, with 46–54 spinules on flexor margin. Endopod of male 1^st^ pleopod extending to 0.8 × exopod length, inflated at distal ¾, pyriform, 1.7 × as long as wide, appendix interna at base of inflated part, short. Appendix masculina of male 2^nd^ pleopod cylindrical, reaching to 0.7 length of endopod, appendix interna reaching to 0.6 length of appendix masculina. Uropodal diaeresis with 13–14 movable spinules. Eggs 1.10 × 0.68 to 1.20 × 0.75 mm in diameter.

###### Description.

Rostrum short, straight, slightly sloping downwards, without distinct postrostral ridge, reaching slightly short of or slightly beyond end of 1^st^ segment of antennular peduncle, occasionally reaching to, rarely beyond end of 2^nd^ segment of antennular peduncle; armed dorsally with 9–18 (mode 13–15) very small teeth, including 1–3 (mode 2) on carapace, ventrally with 1–4 small teeth (mode 2–3). Antennal spine fused with inferior orbital angle. Pterygostomian margin sub-rectangular, armed with an indistinct spine.

Sixth pleomere in male 0.43cl, 1.40 × as long as 5^th^ pleomere, slightly shorter than telson; 6^th^ pleomere in female 0.48cl, 1.38 × as long as 5^th^ pleomere, slightly shorter than telson. Telson 3.0 × as long as wide, with four or five pairs of dorsal spinules and one pair of dorsolateral spinules; posterior margin rounded, lined with four or five pairs of simple setae, lateral pair distinctly longer than intermediate pairs. Pre-anal carina moderately high, lacking spine.

Eyes well developed, anterior corneal margin reaching to 0.6 × length of basal segment of antennular peduncle. Antennular peduncle 0.6 × as long as carapace; basal segment of antennular peduncle longer than combined length of 2^nd^ and 3^rd^ segments, anterolateral angle reaching 0.3 length of 2^nd^ segment, 2^nd^ segment distinctly longer than 3^rd^ segment. Stylocerite reaching 0.7–0.8 length of basal segment of antennular peduncle. Scaphocerite 3.5 × as long as wide, with extension of the distolateral spine reaching end of antennular peduncle.

Mandible with incisor process ending in irregular teeth; molar process truncated. Maxillule lower lacinia broadly rounded; upper lacinia elongate, with a row of 30 distinct spiniform setae on inner margin; palp short. Maxilla distal endite subdivided; palp short; scaphognathite tapering posteriorly with some long, curved setae at posterior end. 1^st^ maxilliped with stout palp. 2^nd^ maxilliped typical of genus, endopod with fused dactylus and propodal segments. 3^rd^ maxilliped reaching to end of antennular peduncle, with ultimate segment slightly longer than penultimate segment.

First four pereiopods with epipod. 1^st^ pereiopod reaching slightly beyond distal end of basal segment of antennular peduncle; merus 1.8–2.1 × as long as broad, as long as carpus; carpus excavated anteriorly, shorter than chela, 1.2–1.5 × as long as high; chela 2.0–2.1 × as long as broad; fingers slightly longer than palm. 2^nd^ pereiopod reaching end of antennular peduncle; merus shorter than carpus, 3.6–4.1 × as long as broad; carpus 1.1–1.2 × as long as chela, 3.9–4.3 × as long as high; chela 2.1–2.3 × as long as broad; fingers 1.3–1.4 × as long as palm. 3^rd^ pereiopod reaching beyond end of antennular peduncle by dactylus; merus stout; propodus straight in females, slightly incurved in males, 2.7–3.0 × as long as dactylus (terminal claw included), 7.2–7.5 × as long as broad, numerous spinules on posterior margin; dactylus terminating in two claws, 4–6 accessory spines on flexor margin, strongly incurved in males. 4^th^ pereiopod similar to 3^rd^ pereiopod in form and length. 5^th^ pereiopod reaching to end of 2^nd^ segment of antennular peduncle, propodus 8.0–9.5 × as long as broad, 2.7–2.8 × as long as dactylus, dactylus 2.9–3.4 × as long as wide (spinules included), terminating in one claw, with 46–54 spinules on flexor margin.

Endopod of male 1^st^ pleopod extending to 0.8 × exopod length, inflated at distal ¾, pyriform , 1.7 × as long as wide, with tiny spinules on distal margin of dorsal surface, appendix interna at base of inflated part, short. Appendix masculina of male 2^nd^ pleopod cylindrical, reaching to about 0.7 length of endopod, inner and distal surface densely lined with long, stout spines, appendix interna reaching to 0.6 length of appendix masculina.

Uropodal diaeresis with 13–14 movable spinules.

Eggs 1.10 × 0.68 to 1.20 × 0.75 mm in diameter.

###### Colour in life.

Body colour varying from translucent to light blue, with darker red-brown spots on dorsal surface and lighter red-brown spots on lateral surface of carapace; pleon usually with several dark red-brown vertical stripes on lower lateral surface, and white star-shaped pigment scattered on whole body (Figure 4A–D). Appendages mostly transparent.

###### Etymology.

*Neocaridinafonticulata* is named after its known habitat, from the Latin root, *fonticulus*, for little spring.

###### Ecological notes.

Specimens of the new species were collected from leaf litter layer of a small stream (Figure 4E, F) next to a spring outlet at a limestone hill. The collection site consists of concretized substrate and banks, representing the headwater of the stream. The water flow is slow, cool temperature (about 25 °C), neutral (pH 7.06–7.16), and with moderately high dissolved oxygen (7.33–7.70 mg/L). The freshwater crabs, *Candidiopotamonrathbuni* (De Man, 1914) and *Geothelphusaferruginea* Shy, Ng & Yu, 1994, were found to be sympatric with this new species. Ovigerous females were found in July.

###### Distribution.

Presently known only from Sheding, Kenting, southern Taiwan.

###### Remarks.

With the short rostrum, *Neocaridinafonticulata* sp. n. is morphologically most similar to the insular Chinese species *Neocaridinazhoushanensis* Cai, 1996, originally described as a subspecies of *N.denticulata*, from Zhoushan Islands of Zhejiang Province. It can be differentiated by the more slender chela of the 1^st^ pereiopod (2.0–2.1 × as long as wide in the new species vs. 1.6–1.7 × in *N.zhoushanensis*; cf. Figures 2A, 3B vs. fig. 10B in Cai 1996); the sexually dimorphic 3^rd^ pereiopods (vs. no sexual dimorphism in *N.zhoushanensis*); the slender dactylus of the 3^rd^ pereiopods (2.9–3.4 × as long as wide (spinules included) vs. 2.8 × in *N.zhoushanensis*; cf. Figures 2C, 3D vs. fig. 10E in Cai 1996), the spination of the dactylus of 5^th^ pereiopods (with 46–54 spinules on flexor margin vs. 35–43 in *N.zhoushanensis*); and the shorter appendix interna on the male 2^nd^ pleopod, which reaches to 0.6 length of appendix masculina (vs. 0.7 in *N.zhoushanensis*; cf. Figure 2H vs. fig. 9F in Cai 1996).

With the relatively short rostrum, *Neocaridinafonticulata* sp. n. morphologically resembles two Taiwanese species, *N.saccam* Shih & Cai, 2007 and *N.ketagalan* Shih & Cai, 2007. It differs from *N.saccam* (cf. Shih and Cai 2007) by the shorter rostrum (falling slightly short of or reaching slightly beyond the end of the 1^st^ segment of the antennular peduncle vs. reaching the midlength of the 2^nd^ segment of the antennular peduncle or nearly reaching beyond it in *N.saccam*; cf. Figures 1A, 3A vs. figs 2A, 4A in Shih and Cai 2007); the more slender merus of the 1^st^ pereiopod (1.8–2.0 × as long as high vs. 1.4–1.7 × in *N.saccam*; Figures 2A, 3B vs. figs 3A, 4B in Shih and Cai 2007); the more slender carpus of the 2^nd^ pereiopod (3.9–4.3 × as long as high vs. 3.6–3.8 × in *N.saccam*; Figures 2B, 3C vs. figs 3B, 4C in Shih and Cai 2007); the more slender male 1^st^ pleopod (1.7 × as long as broad vs. 1.4 × in *N.saccam*; Figure 2G vs. fig. 3G in Shih and Cai 2007); and the shorter endopod of the male 1^st^ pleopod (0.8 × length of exopod vs. 0.9 × in *N.saccam*; Figure 2G vs. fig. 3G in Shih and Cai 2007).

**Figure 5.**
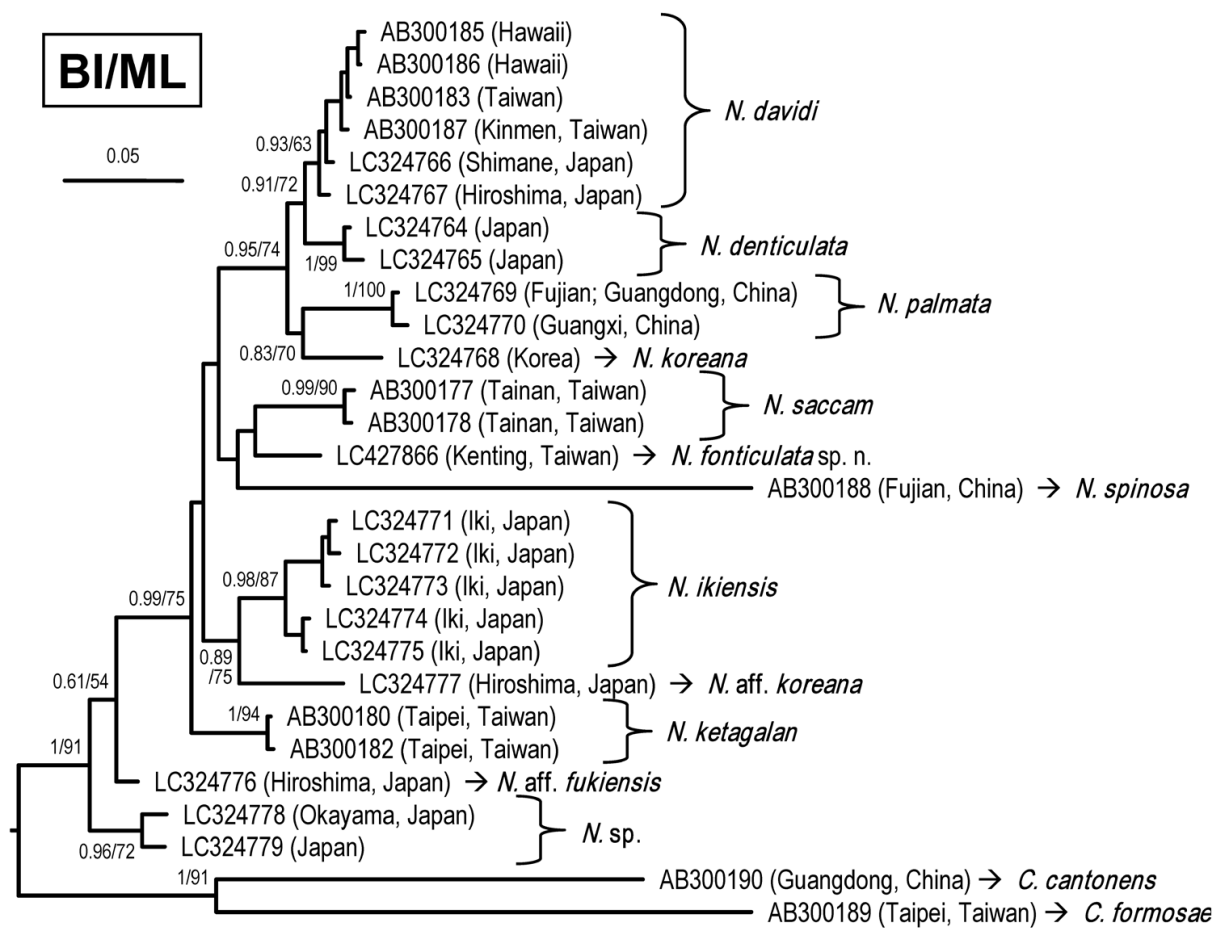
Bayesian inference (BI) tree of species of *Neocaridina* from East Asia and outgroups based on COI gene. Support values at the nodes represent posterior probability and bootstrap values for BI and maximum likelihood (ML), respectively.

*Neocaridinafonticulata* sp. n. can be separated from *N.ketagalan* (cf. Shih and Cai 2007) by its shorter rostrum (reaching from slightly short of to slightly beyond end of 1^st^ segment of antennular peduncle vs. reaching the middle or end of the 2^nd^ segment of the antennular peduncle; Figures 1A, 3A vs. figs 5A, 6A in Shih and Cai 2007). It also differs from *N.ketagalan* (cf. Shih and Cai 2007) by the slender male 1^st^ pleopod (1.7 × as long as broad vs. 1.4 × in *N.ketagalan*; Figure 2G vs. fig. 5J in Shih and Cai 2007); the male 2^nd^ pleopod appendix masculina being half the endopod length (vs. 0.7 × in *N.ketagalan*; Figure 2H vs. fig. 5K in Shih and Cai 2007); and the appendix interna of the male 2^nd^ pleopod being relatively longer, reaching to 0.7 × length of appendix masculine (vs. 0.6 × in *N.ketagalan*; Figure 2H vs. fig. 5K in Shih and Cai 2007).

With its relatively short rostrum, *Neocaridinafonticulata* sp. n. morphologically also resembles the recently described Japanese species *Neocaridinaikiensis* Shih, Cai, Niwa & Nakahara, 2017. It can be differentiated from the latter by its shorter rostrum (reaching from slightly short of to slightly beyond the end of the 1^st^ segment of antennular peduncle vs. reaching slightly short of to distinctly beyond the end of the 2^nd^ segment of antennular peduncle; cf. Figures 1A, 3A vs. figs 2A, 4A in Shih et al. 2017). The propodus and dactylus of the 3^rd^ pereiopod of the new species displays sexual dimorphism (vs. no sexual dimorphism in *N.ikiensis*); the male 2^nd^ pleopod appendix masculina is 0.7 × endopod length (vs. 0.5 × in *N.ikiensis*; Figure 2H vs. fig. 3I in Shih et al. 2017); and the appendix interna of the male 2^nd^ pleopod is relatively shorter (reaching to 0.6 length of appendix masculina vs. 0.8 in *N.ikiensis*; Figure 2H vs. fig. 3J in Shih et al. 2017).

With the relatively slender endopod of the male 1^st^ pleopod, the new species is similar to *N.koreana* Kubo, 1938. It can be separated from the latter by the relatively shorter rostrum, which mostly reaches to or slightly beyond the end of the 1^st^ segment of antennular peduncle vs. almost reaching to or slightly beyond antennular peduncle in *N.koreana* (cf. Kubo 1938); and the fewer ventral rostral teeth (2–6 teeth, mode 2–4 vs. 4–6, average 5.6 in *N.koreana* (cf. Kubo 1938)).

## DNA analyses and discussion

A total of four specimens from Sheding, Kenting, were used in the molecular phylogenetic analysis. A 658-bp segment of COI was amplified, resulting in one haplotype (accession number LC427866). Based on the COI haplotypes, the phylogenetic tree was reconstructed using BI analysis, with the support values from the BI and ML analyses shown in Figure 5. Specimens assigned to *Neocaridinafonticulata* sp. n. formed a clade distinct from other species. The pairwise nucleotide divergences with the K2P distance and bp differences of haplotypes are shown in Table 1. The minimum K2P interspecific divergences between *N.fonticulata* sp. n. and *N.ketagalan* and *N.saccam* are 5.42% and 5.43% respectively, which are close to or larger than the values between species of *N.davidi*, *N.denticulata*, *N.koreana*, and *N.palmata* (from 2.17% to 5.56%; Table 1). Consequently, the establishment of the new taxon is warranted.

**Table 1.** Matrix of percentage pairwise nucleotide divergences (lower left) and mean number of differences (upper right) based on COI within and between some species of *Neocaridina* from East Asia. Values of range are shown in parentheses.

	Intraspecific		Interspecific						
	Nucleotide divergence	Mean nucleotide difference	* N. davidi *	* N. denticulata *	* N. koreana *	* N. palmata *	*N.fonticulata* sp. n.	* N. ketagalan *	* N. saccam *
*** N. davidi ***	0.67	4.39		17.75	29.88	30.63	36.5	44.46	45.67
	(0–1.54)	(0–10)		(14–22)	(28–32)	(28–34)	(35–39)	(41–48)	(42–50)
*** N. denticulata ***	0.46	3	2.77		31.5	33.17	38.5	48.83	48.83
	(0–0.77)	(0–5)	(2.17–3.46)		(30–33)	(31–36)	(36–41)	(46–52)	(47–51)
*** N. koreana ***	0 (0)	0 (0)	4.73	5		35.67	46	46.33	52.33
			(4.42–5.07)	(4.75–5.25)		(35–37)	(46–46)	(46–47)	(52–53)
*** N. palmata ***	0.41	2.67	4.83	5.26	5.67		47 (47)	48.33	53 (53–53)
	(0–0.61)	(0–4)	(4.4–5.39)	(4.9–5.73)	(5.56–5.89)			(48–49)	
***N.fonticulata* sp. n.**	0 (0)	0 (0)	5.82	6.16	7.41	7.56		34.67	34.33
			(5.57–6.24)	(5.74–6.59)	(7.41)	(7.56–7.56)		(34–35)	(34–35)
*** N. ketagalan ***	0.1	0.67	7.17	7.94	7.49	7.82	5.53		38
	(0–0.15)	(0–1)	(6.58–7.78)	(7.44–8.5)	(7.44–7.61)	(7.76–7.93)	(5.42–5.58)		(37–39)
*** N. saccam ***	0.31	2	7.39	7.94	8.51	8.62	5.49	6.08	
	(0–0.46)	(0–3)	(6.75–8.14)	(7.62–8.32)	(8.45–8.63)	(8.62–8.62)	(5.43–5.6)	(5.91–6.25)	

The discovery of the new species increases the number of *Neocaridina* species in Taiwan to four, i.e., *N.davidi*, *N.saccam*, *N.ketagalan*, and *N.fonticulata* sp. n. (Shih and Cai 2007, Shih et al. 2017). While the common species, *N.davidi*, is distributed in both western and eastern sides of Taiwan Island, as well as the offshore islands, Penghu, Siaoliouciou and Kinmen (Shih and Cai 2007, Shih et al. 2017; unpublished data), the distributional range of the other three endemic species is narrower. *Neocaridinaketagalan* is distributed in northern Taiwan, *N.saccam* is limited in southwestern Taiwan, and *N.fonticulata* sp. n. is presently known only from Kenting. Previous molecular studies on aquatic organisms, including the freshwater crab *Candidiopotamonrathbuni* (De Man, 1914) and frog *Hylaranalatouchii* (Boulenger, 1899) (= *Sylviranalatouchii*) (Shih et al. 2006, Jang-Liaw et al. 2008), have shown the populations in Hengchun Peninsula to be closely related to the populations in eastern Taiwan due to the weak isolation effect of the lower mountains. It is expected that *Neocaridinafonticulata* sp. n. will be found in eastern Taiwan as well.

## Supplementary Material

XML Treatment for Neocaridina fonticulata

## References

[B1] Cai Y (1996) A revision of the genus *Neocaridina* (Crustacea: Decapoda: Atyidae). Acta Zootaxonimica Sinica 21: 129–160. [In Chinese]

[B2] De Grave S Fransen CHJM (2011) Carideorum catalogus: the recent species of the dendrobranchiate, stenopodidean, procarididean and caridean shrimps (Crustacea: Decapoda). Zoologische Mededelingen, Leiden 85: 195–588.

[B3] Folmer O Black M Hoeh W Lutz R Vrijenhoek R (1994) DNA primers for amplification of mitochondrial cytochrome c oxidase subunit I from diverse metazoan invertebrates. Molecular Marine Biology and Biotechnology 3: 294–299. 7881515

[B4] Jang-Liaw NH Lee TH Chou WH (2008) Phylogeography of *Sylviranalatouchii* (Anura, Ranidae) in Taiwan. Zoological Science, Tokyo 25: 68–79. 10.2108/zsj.25.68 18275248

[B5] Kimura M (1980) A simple method for estimating evolutionary rates of base substitutions through comparative studies of nucleotide sequences. Journal of Molecular Evolution 16: 111–120. 10.1007/BF01731581 7463489

[B6] Kubo I (1938) On the Japanese atyid shrimps. Journal of the Imperial Fisheries Institute, Tokyo 33: 67–100.

[B7] Liang XQ (2004) Fauna Sinica. Invertebrata: Crustacea: Decapoda: Atyidae. Science Press, Beijing, 375 pp. [In Chinese]

[B8] Naruse T Shokita S Cai Y (2006) *Neocaridinairiomotensis*, a new species of land-locked freshwater shrimp (Crustacea: Decapoda: Atyidae) from Iriomote island, southern Ryukyus, Japan. Proceedings of the Biological Society of Washington 119: 25–31. 10.2988/0006-324X(2006)119[25:NIANSO]2.0.CO;2

[B9] Nylander JAA (2005) MrModeltest version 2.2. Evolutionary Biology Centre, Uppsala Univ., Uppsala.

[B10] Ronquist F Huelsenbeck JP van der Mark P (2005) MrBayes 3.1 manual. http://mrbayes.csit.fsu.edu/manual.php

[B11] Ronquist F Teslenko M van der Mark P Ayres DL Darling A Höhna S Larget B Liu L Suchard MA Huelsenbeck JP (2012) MRBAYES 3.2: efficient Bayesian phylogenetic inference and model choice across a large model space. Systematic Biology 61: 539–542. 10.1093/sysbio/sys029 22357727 PMC3329765

[B12] Shih HT Cai Y (2007) Two new species of land-locked freshwater shrimp genus *Neocaridina* Kubo, 1938 (Decapoda: Caridea: Atyidae) from Taiwan, with notes on the speciation within Taiwan Island. Zoological Studies 46: 680–694.

[B13] Shih HT Cai Y Niwa N Nakahara Y (2017) A new species of land-locked freshwater shrimp of the genus *Neocaridina* (Decapoda: Caridea: Atyidae) from Iki Island, Kyushu, Japan. Zoological Studies 56: 30. 10.6620/ZS.2017.56-30 PMC6517772 31966229

[B14] Shih HT Hung HC Schubart CD Chen CA Chang HW (2006) Intraspecific genetic diversity of the endemic freshwater crab *Candidiopotamonrathbunae* (Decapoda, Brachyura, Potamidae) reflects five million years of geological history of Taiwan. Journal of Biogeography 33: 980–989. 10.1111/j.1365-2699.2006.01472.x

[B15] Stamatakis A (2006) RAxML-VI-HPC: maximum likelihood-based phylogenetic analyses with thousands of taxa and mixed models. Bioinformatics 22: 2688–2690. 10.1093/bioinformatics/btl446 16928733

[B16] Tamura K Peterson D Peterson N Stecher G Nei M Kumar S (2011) MEGA5: Molecular Evolutionary Genetics Analysis using maximum likelihood, evolutionary distance, and maximum parsimony methods. Molecular Biology and Evolution 28: 2731–2739. 10.1093/molbev/msr121 21546353 PMC3203626

